# Synthesis, characterization and modelling of zinc and silicate co-substituted hydroxyapatite

**DOI:** 10.1098/rsif.2015.0190

**Published:** 2015-07-06

**Authors:** Robert J. Friederichs, Helen F. Chappell, David V. Shepherd, Serena M. Best

**Affiliations:** 1 Department of Materials Science and Metallurgy, University of Cambridge, 27 Charles Babbage Road, Cambridge CB3 0FS, UK; 2 Department of Archaeology and Anthropology, University of Cambridge, Downing Street, Cambridge CB2 3DZ, UK; 3 MRC Human Nutrition Research, 120 Fulborn Road, Cambridge CB1 9NL, UK

**Keywords:** hydroxyapatite, calcium phosphate, zinc, silicon, silicate, modelling

## Abstract

Experimental chemistry and atomic modelling studies were performed here to investigate a novel ionic co-substitution in hydroxyapatite (HA). Zinc, silicate co-substituted HA (ZnSiHA) remained phase pure after heating to 1100°C with Zn and Si amounts of 0.6 wt% and 1.2 wt%, respectively. Unique lattice expansions in ZnSiHA, silicate Fourier transform infrared peaks and changes to the hydroxyl IR stretching region suggested Zn and silicate co-substitution in ZnSiHA. Zn and silicate insertion into HA was modelled using density functional theory (DFT). Different scenarios were considered where Zn substituted for different calcium sites or at a *2b* site along the *c*-axis, which was suspected in singly substituted ZnHA. The most energetically favourable site in ZnSiHA was Zn positioned at a previously unreported interstitial site just off the *c*-axis near a silicate tetrahedron sitting on a phosphate site. A combination of experimental chemistry and DFT modelling provided insight into these complex co-substituted calcium phosphates that could find biomedical application as a synthetic bone mineral substitute.

## 1. Introduction

The chemical similarity of synthetic hydroxyapatite (HA) Ca_10_(PO4)_6_(OH)_2_ and natural bone mineral has led to its use as a bone grafting material. Synthetic HA is well known for its ability to bond with bone tissue, but it is limited by a lower solubility compared with other popular orthopaedic implant materials such as tricalcium phosphate (TCP) or silica-based bioglass [1,2]. Many researchers have considered ionic substitutions in synthetic HA as a means to enhance the bioactivity of HA in bone-contacting applications [3]. The apatite structure of HA (*P6*_3_*/m* space group) allows for ionic substitution or interstitial site insertion depending on the substituting ion, thermodynamic formation energies, kinetics of ion exchange and reaction environment. This paper refers to the six Ca atomic sites Posner termed ‘hydroxyl-associated’ as CaII. These sites are arranged in equilateral triangles along the *c*-axis, spaced at half a unit cell apart perpendicular to the basal plane (*a*, *b* axes) as CaII. The remaining four ‘columnar’ Ca atomic sites are referred to as CaI. For further reading and illustrations of HA crystallography, the seminal work of Posner is recommended [4].

Silicon plays an important role in connective tissue health as demonstrated in studies by Carlisle *et al.* [5] and Jugdaohsingh *et al.* [6], although its biochemical role remains unclear. Subsequently, Si-substituted HA (SiHA) has found use as a successful implantable orthopaedic material. Many of the biological studies involving SiHA have focused on materials with 0.8–1.5 wt% Si. For example, markers of osteoblast activity were enhanced during *in vitro* culture on SiHA 0.8 wt% [7], and increased bone formation was found in a porous SiHA 0.8 wt% scaffold at three and six weeks in a lapine model compared with HA and both SiHA 0.4 and 1.5 wt% [8]. Furthermore, organized collagen fibrils were formed on 1.5 wt% SiHA compared with disordered fibrils on HA, after implantation in lapine models [9]. The mechanisms responsible for the success of SiHA are still under investigation [10], but one study suggested that the increased concentration of triple-point defects in SiHA compared with HA might collectively increase the solubility of SiHA [11]. Hydroxyl vacancy formation, suggested by Gibson *et al.* remains the most widely accepted charge balance mechanism for silicate ion substitution on a phosphate site in HA [12]. This has been shown to vary, however, with the method of synthesis and heat-treatment temperature [13–15]. Silicon (Si) substitution limits are typically approximately 2 wt% Si before phase decomposition occurs with heat treatment, although this can vary depending on the synthesis method and source of Si [14–16].

While high levels of Zn are known to be cytotoxic [17], low substitution levels in calcium phosphates (CaPs) have been investigated for their potential to stimulate bone formation, act as an anti-microbial and slow osteoclast (OC) resorption. Yamaguchi *et al.* found that solubilized zinc (zinc sulfate) between 10^−6^ and 10^−3^ M increased bone alkaline phosphatase, 10^−6^ and 10^−4^ M increased bone collagen and 10^−4^ M increased bone calcium levels in murine calvarial bone culture [17]. Stanić *et al.* [18] produced ZnHA that inhibited the growth of bacteria (*Escherichia coli* and *Staphylococcus aureus*) and yeast (*Candida albicans*) *in vitro*. Additionally, lapine OCs showed reduced volume resorption on Zn β-TCP (0.63 wt%) compared with β-TCP after 24 h *in vitro* [19], and Shepherd *et al.* [20] showed reduced human OC resorption on ZnHA (0.4 wt%) *in vitro* at 21 days compared with HA. However, the mechanism of Zn substitution into HA is not clear and the findings in the literature are ambiguous. Zn^2+^ ions were initially assumed to substitute isoelectronically into a Ca^2+^ site vacancy in HA, and modelling studies deemed this possible, with an energetic preference for hexagonal CaII atomic sites [21,22]. Later studies by Gomes *et al.* using X-ray diffraction (XRD), neutron diffraction and Raman spectroscopy suggested that Zn was present at a *2b* [0,0,0] atomic site in the hydroxyl channel, although it should be noted that their ZnHA (1.8–13 wt% Zn) also contained β-TCP [23]. Hu *et al.* used atomic modelling and advanced X-ray techniques to investigate the effect of Zn concentration on substitution location. They found that at lower substitution levels (0.1 mol%, Zn/(Zn + Ca)) interstitial insertion may be favoured, and at higher Zn levels (0.5 and 1 mol%) CaII site substitution was preferred [24].

Co-substitution of ions in HA has the potential to combine several desirable characteristics of singly substituted HA. Many ionic substitutions have appeared in the literature, but selecting ions for co-substitution must be performed carefully. Ionic charge balance, atomic substitution sites, phase stability and possible biological impact must be considered. Zn and Si co-substitution in HA could potentially enhance bioactivity and act as an anti-microbial agent. Wei *et al.* performed a qualitative direct contact cytotoxicity test that involved placing ZnSi-TCP particles on confluent human ovarian carcinoma cells (SKOV3) for 24 h with a positive (toxic) control induced by ZnO and a negative control with no particle addition [25]. The ZnSi-TCP did not cause the cells to recede from the particles indicating lack of a ‘toxic’ response, but no definitive toxicity assays were performed and bone derived cells were not considered. This leaves room for further study of Zn and Si co-substituted CaPs.

The synthesis and characterization of phase pure ZnSiHA are discussed in this study for the first time. A range of ZnSiHA compositions with different Zn and Si amounts, similar to those produced by Shepherd & Best [26] and Gibson *et al.* [27], were produced and characterized here using XRD, Fourier transform infrared (FTIR) spectroscopy and X-ray fluorescence (XRF). Atomic modelling was performed to determine energetically favourable locations for Zn and Si in the HA lattice.

## 2. Material and methods

### 2.1. Materials synthesis

ZnSiHA powders were synthesized using a technique based on the cumulative efforts of Jarcho and Akao (HA) [28,29], Gibson *et al.* (SiHA) [12] and Shepherd & Best (ZnHA) [26]. Stoichiometric HA, SiHA and ZnHA were also produced, using similar methods described in the aforementioned papers. The charge balance mechanism in equation (2.1) was assumed for the calculation of reagents for ZnSiHA, and the expected Ca + Zn/P + Si ratios were equal to that of stoichiometric HA at 1.667. A summary of the compositions and expected molar ratios are listed in table 1. Specific compositions of ZnSiHA will now be referred to in the text by those theoretical Zn*_x_*Si*_y_* amounts listed in table 1.

**Table 1.** Calculated empirical substitution amounts with expected weight percentages for atoms based on a Ca + Zn/P + Si substitution suggested in equation (2.1). Phase purity was lost upon heating to 1100°C for samples in the rows below Zn_0.1_Si_0.5_HA.

	Zn	Si	Zn	Si	theoretical
sample	*x*	*y*	wt%	wt%	Ca/P
HA	0	0	0	0	1.667
SiHA	0.3	0	0	0.84	1.750
SiHA	0.5	0	0	1.5	1.812
ZnHA	0.062	0	0.4	0	1.656
ZnHA	0.101	0	0.66	0	1.650
ZnSiHA	0.062	0.301	0.4	0.85	1.744
ZnSiHA	0.061	0.533	0.4	1.5	1.820
ZnSiHA	0.1	0.302	0.65	0.85	1.737
ZnSiHA	0.1	0.533	0.66	1.5	1.811
ZnSiHA	0.2	0.302	1.3	0.85	1.720
ZnSiHA	0.3	0.302	1.8	0.84	1.702
ZnSiHA	0.2	0.534	1.2	1.5	1.793
ZnSiHA	0.3	0.535	2	1.5	1.775
ZnSiHA	0.1	1	0.7	2.8	1.980
ZnSiHA	0.1	2	0.7	5.8	2.476

Calcium, phosphorus, zinc and silicon precursors were combined in an aqueous reaction vessel at an ambient atmosphere. The source of calcium was CaCO_3_ (Sigma Aldrich, ACS reagent grade 239216, UK) that was decarburized in a furnace to form high-purity CaO, which was then hydrated in deionized water (2 l water to 1 mol Ca) to form Ca(OH)_2_. ZnNO_3_•6H_2_O (Sigma Aldrich reagent grade 98% 228737, UK) and tetraethylorthosilicate (TEOS, Sigma Aldrich ≥99% 86578, UK) were subsequently added to the calcium-containing solution. H_3_PO_4_ (85.4% v/v, Fisher Scientific, UK) was diluted with deionized water (2 : 1, l water to mol Ca) in a separate container, and then added to the calcium-containing solution at a rate of 5 ml min^−1^. The pH of the solution was kept above 10.5 through the addition of 35% NH_3_ (*aq*) solution (Fisher Scientific analytical reagent grade). Upon complete addition of the phosphoric acid solution to the calcium solution, the mixture was stirred for 2 h, and then aged overnight. Test batches were made with 0.09 mol of Ca, but this was later scaled up to 0.25 mol Ca. Dried filter cake was ground in a mortar and pestle, and then fired at temperatures between 1000 and 1200°C.2.1
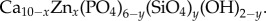


### 2.2. Characterization methods

#### 2.2.1. X-ray diffraction

The phase purity of ZnSiHA particles heat-treated to 1100°C was investigated with XRD over a range of 25–50° 2*θ*. Powder XRD scans were performed using a Phillips PW1050 diffractometer (PANalytical, NL) with monochromatic Cu Kα X-rays, a 0.05 step size and a sweep rate of 1° 2*θ* min^−1^. Phillips HighScore plus software was used to identify phases in the heat-treated CaP powders. ICDD (International Centre for Diffraction Data) powder diffraction files of HA (09-0432), α-TCP (29-0359), β-TCP (70-2065), CaO (37-1497), ZnO (89-7102), tetracalcium phosphate (25-1137), calcium carbonate CaCO_3_ (calcite) (85-1108), calcium silicate Ca_2_SiO_4_ (86-0401), silicocarnotite Ca_5_(PO_4_)_2_SiO_4_ (40-0393), CaZn_2_(PO_4_)_2_•2H_2_O (35-0495) and Zn_3_(PO_4_)_2_•4H_2_O (33-1474) were compared to observed diffraction patterns.

XRD spectra were used in lattice parameter refinement of ZnSiHA heated to 1100°C. Scans were performed using a Phillips X'Pert PW 3020 instrument with monochromatic Cu Kα X-rays over a range of 10–110° 2*θ*. A step size of 0.025° and a sweep rate of 0.15° 2*θ* min^−1^ were used. Lattice parameters were refined using Phillips HighScore plus software based on Rietveld code from Wiles & Young [30]. Starting parameters from Kay and Posner's X-ray data with a space group of *P6*_3_/*m* were used [31]. Background parameters, scale factor, unit cell parameters, sample displacement error, profile parameters (U,W) and peak widths (pseudo-Voigt) were refined. Rwp, goodness of fit (GOF/*χ*^2^) and difference plots were followed for improvements in fit. The text refers to unit cell axes as parameters *a*, *b* and *c*.

#### 2.2.2. Fourier transform infrared spectroscopy

FTIR spectroscopy of heat-treated ZnSiHA (1100°C) was performed on a Bruker Tensor 27 with a resolution of 4 cm^−1^. Sample particles and KBr (IR grade) were dried overnight at 110°C to avoid water contamination at higher frequencies, and then pellets were prepared for transmittance FTIR. Scans were taken over the range of 400 to 4000 cm^−1^ and the values were averaged over 128 scans.

#### 2.2.3. X-ray fluorescence

XRF was performed on as-precipitated ZnSiHA samples by AMG Superalloys Ltd (UKAS 1091, UK accredited) as part of the OXI package with minimum detection limits of 0.05 wt% for elemental oxides including CaO, ZnO, SiO_2_, P_2_O_5_, Na_2_O, MgO, Al_2_O_3_, K_2_O, TiO_2_, Mn_3_O_4_, V_2_O_5_, Cr_2_O_3_, Fe_2_O_3_, BaO, ZrO_2_ and SrO.

### 2.3. Computational modelling

The plane wave density functional theory (DFT) code CASTEP [32] was used to predict the most thermodynamically stable environment for the co-substitution of zinc and silicon in the HA unit cell. The generalized gradient approximation and PBE exchange-correlation functional were employed [33], with a convergence tested kinetic cut-off energy of 430 eV. The Brillouin zone [34] was sampled with a *k*-point grid of 3 × 3 × 3. Convergence tolerances for energy change, maximum force, maximum stress and maximum displacement were set to 1 × 10^−5^ eV atom^−1^, 0.03 eV Å^−1^, 0.05 GPa and 0.001Å, respectively. Ultrasoft pseudopotentials [35] were employed for all elements. A test model of a single 44-atom hexagonal HA unit cell was used as previously described by the authors [22]. The optimized structure produced lattice parameters of *a* = 9.477, *b* = 9.478 and *c* = 6.851.

Formation energies were used to test the stability of the ion substitutions in the unit cell. Chemical potentials for calcium, silicon, zinc and phosphorus were calculated from various sources and sinks and the lowest values chosen to proceed: metallic calcium, silicon, metallic zinc and monoclinic phosphorus, respectively. For a double substitution of silicon and zinc, the formation energy can be calculated from equation (2.2), where *E*_ZnSiHa_ is the energy of the doubly substituted HA unit cell, *E*_HA–OH_ the energy of a HA unit cell with one hydroxyl ion removed and *μ*_P_, *μ*_Ca_, *μ*_Si_ and *μ_Z_*_n_ the chemical potentials of phosphorus, calcium, silicon and zinc, respectively. All other formation energies were calculated in a similar manner.2.2
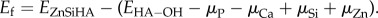


## 3. Results and discussion

### 3.1. X-ray diffraction analysis

#### 3.1.1. X-ray diffraction phase analysis

**Figure 1.**
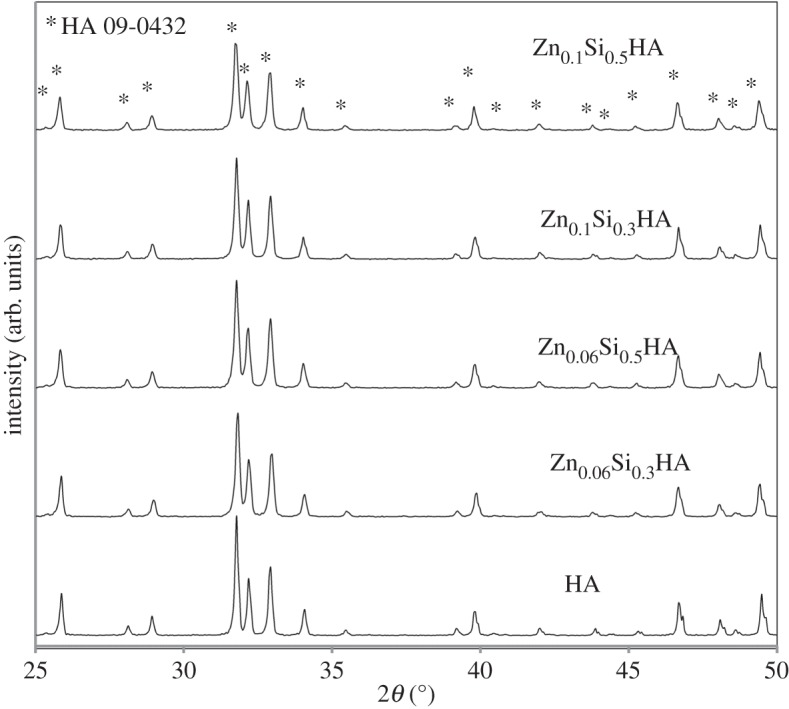
XRD traces of phase pure ZnSiHA particles heated to 1100°C. Asterisks (*) indicate the HA phase (ICDD card 9-432).

ZnSiHA was phase pure at four different Zn and Si substitution levels (table 1 and figure 1) that were intended to correspond to previously synthesized singly substituted phase pure ZnHA (approx. 0.4 and approx. 0.6 wt% Zn) [26] and SiHA (0.84 and 1.5 wt% Si) [27]. The most highly substituted phase pure sample after heating to 1100°C was Zn_0.1_Si_0.5_HA. As Zn increased (Zn_0.2_Si_0.3_HA and Zn_0.3_Si_0.3_HA), the α-TCP phase appeared. Silicocarnotite appeared alongside α-TCP and HA phases in Zn_0.3_Si_0.5_HA, and then a biphasic HA/silicocarnotite mixture was present in Zn_0.1_Si_2_HA, where the Si amount was dramatically increased (theoretical 5.8 wt% Si). Si substitution in CaPs is already known to stabilize the α-TCP phase below its normal formation temperature of 1125°C [36] and Gomes *et al.* reported silicocarnotite formation in Si_1.0_HA heated to 1100°C [37]. Interestingly, Zn has been shown to stabilize the β-TCP phase at elevated temperatures (600–1100°C) [23,38], but this was not observed in the ZnSiHA produced here. Our study also shows that previously observed substitution limits for Zn (0.58 wt% (*x* = 0.1) observed by Shepherd *et al.*) and Si (1.6 wt% (*y* = 0.5) observed by Gibson *et al.*) have not increased as a result of co-substitution using this wet chemical precipitation method.

All of the ZnSiHA samples were heated to 1100°C to crystallize the sample for diffraction experiments and encourage Zn substitution. A thermal study of Zn insertion into the HA lattice by Gomes *et al.* (1.6, 3.2 and 6.1 wt% Zn) showed that the majority of Zn did not enter the lattice until heated to over 1000°C [38]. Equally, silicate may not completely substitute in HA after precipitation due to ambient carbonate substitution from the atmosphere and subsequent Si(OH)_4_ formation, which upon heating above approximately 900°C liberates carbonate and substitutes any remaining Si as silicate [15]. The phase purity present in some of the ZnSiHA samples certainly suggested Zn and Si co-substitution occurred, otherwise the stoichiometry would have deviated significantly, resulting in the loss of the HA phase. However, changes in the HA lattice parameters, FTIR and XRF were used to follow ionic insertion of Zn and Si more closely.

An emphasis on phase pure materials was made to set a precedent for future studies investigating the physico-chemical and biological nature of ZnSiHA. Substituting ions can distribute unequally into secondary phases or in the case of Si, stabilize a secondary phase (TCP) [39] creating an increasingly complex material to characterize when investigating co-ionic substitutions. Secondary phases alter calcium solubility, which could confuse biological comparisons between materials. Whether a cellular response is due to the ionic substitutions in HA or a more soluble secondary phase, such as CaO or TCP, confounds the investigation. Attributing a bioactive response of bone to a particular ion substitution in HA is extremely difficult and it is for this reason care has been taken to ensure the phase purity of ZnSiHA as a first step in understanding the effect and, ultimately, the function of a particular co-substitution. Determining the mechanisms of bioactive responses during further development phases of the materials relies on careful manufacture and characterization at this stage.

#### 3.1.2. Rietveld refinement of lattice parameters

**Figure 2.**
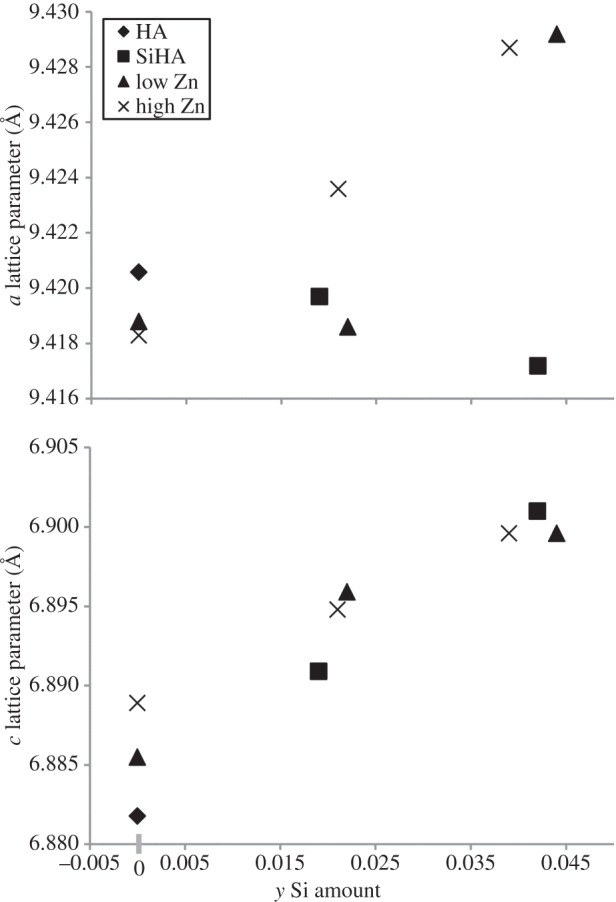
Lattice parameters with respect to silicon amount as measured by XRF.

The lattice parameters of ZnSiHA differed from HA, singly substituted ZnHA and SiHA, suggesting co-substitution (table 2). Plots of *a* and *c* parameters with respect to Zn and Si amount are shown in figure 2. In the case of SiHA, the *a* parameter contracted and the *c* parameter expanded compared with HA, which was similar to the trends observed by Gibson *et al.* [12], who also used a wet chemical precipitation method. The *c* parameter typically increases with Si substitution given the relatively larger ionic radius of Si compared with P, and relative charge difference of silicate on the phosphate site [40]. The *a* parameter and unit cell volumes were found to vary widely in the literature. This variation in the *a* parameter is likely due to the many different synthesis methods and subsequent heat treatment environments used in making SiHA. Marchat *et al.* suggested that the *a* parameter variation in SiHA likely results from OHA (oxyhydroxyapatite) formation, which was shown to slightly reduce the *a* parameter length [14,41].

**Table 2.** Refinement results from XRD data. Lattice parameters, unit cell volumes, indices of fit and error (s.d.) calculated using HighScore plus software.

	angstroms (Å)		volume (Å^3^)	agreement indices		
sample	*a* = *b*	*c*	*v*	GOF	Rwp %	Rexp %
HA	9.4206(1)	6.8818(1)	528.92	5.12	16.7	7.37
Si_0.3_HA	9.4197(1)	6.8909(1)	529.52	4.11	14.7	7.23
Si_0.5_HA	9.4172(1)	6.9010(1)	530.01	3.90	15.3	7.71
Zn_0.06_HA	9.4188(1)	6.8855(1)	529.00	3.93	15.8	7.97
Zn_0.1_HA	9.4183(1)	6.8889(1)	529.20	3.53	17.4	9.26
Zn_0.06_Si_0.3_HA	9.4186(1)	6.8959(1)	529.78	3.20	13.9	7.76
Zn_0.06_Si_0.5_HA	9.4292(1)	6.8996(1)	531.26	3.00	13.9	8.02
Zn_0.1_Si_0.3_HA	9.4236(1)	6.8948(1)	530.20	4.20	15.0	7.28
Zn_0.1_Si_0.5_HA	9.4287(1)	6.8996(1)	531.20	3.90	13.6	6.88

ZnHA had similar lattice distortions compared with SiHA, although with a lesser magnitude (table 2). Gomes *et al.* reported sinusoidal fluctuations in both HA lattice parameters between 500 and 1100°C suggesting the importance of heat treatment for Zn insertion into HA. At 1100°C, the *a* parameter decreased and the *c* parameter increased as was found for the ZnHA in this study (table 2). These lattice parameter changes may suggest a Zn insertion mechanism at the hexagonal axis *2b* site where Zn pushes the close-by O4 (hydroxyl-O) atoms apart, expanding the *c* direction and distorting the nearby phosphate tetrahedron by attracting O3 (phosphate-O) atoms and shrinking the *a* direction [23]. However, Tang *et al.* also proposed that the *a* parameter contraction could also imply Zn substitution for Ca_2_ where Zn and hydroxyl ions move towards each other [22].

ZnSiHA *a* lattice parameters did not follow the decreasing trends observed for the singly substituted SiHA and ZnHA. Aside from Zn_0.06_Si_0.3_HA, ZnSiHA *a* parameters increased with Si amount (figure 2). ZnSiHA *c* parameters also increased with increasing silicate amount (figure 2) and stayed relatively constant with increasing Zn content. ZnSiHA had distortions in the *c* direction of a magnitude similar to SiHA. Contrary to both singly substituted apatites, at high silicate amounts in ZnSiHA *a* lattice parameter considerably increased. The *a* parameters showed no clear trends for ZnSiHA, which could imply changes in Zn substitution mechanism as previously discussed or a lattice expansion unique to the Zn/Si distortion. These scenarios both support Zn and silicate insertion into the HA lattice. Our modelling results (§3.4) show an increase in both *a* and *c* parameters for the *2b* model and an increase in the *c* parameter with a very slight decrease in the *a* parameter (0.2%) for the calcium substitution position.

X-ray scattering similarities between P/Si and Ca/Zn, multiplicity (per unit cell) of different atomic sites and low amounts of Zn (less than 0.6 wt%) prevented successful Rietveld refinement of the atomic site for Zn in ZnSiHA. Attempts to refine atomic positions (CaI, CaII, P, OI-IV and Zn), thermal parameters (*B*_iso_; 1 for Ca and Zn, 1 for P, and 1 for O) and Zn occupancies from XRD patterns failed. Refinements where Zn was positioned in ZnSiHA at either the CaI, CaII or *2b* [0,0,0] atomic sites or the two interstitial atomic sites generated from DFT modelling (§3.4) were unsuccessful. Si was not input into the refinement model due to the lack of X-ray contrast between P and Si. The X-ray contrast of Ca and Zn (approx. two-thirds scattering ratio) theoretically allows for the identification of these atoms when there is a sufficient wt% of Zn, but this was not observed in our approximately 0.4 or approximately 0.6 wt% (*x* = 0.06 and 0.1, respectively) Zn-containing samples. Gomes *et al.* detected Zn at the *2b* site in Zn_0.25_HA, Zn_0.5_HA and Zn_1.0_HA using XRD, but the *2b* site multiplicity of 2 (compared to 4 for CaI or 6 for CaII) coupled with higher Zn amounts explains their successful refinements. We did not consider larger Zn amounts similar to those used by Gomes *et al.* because of (i) HA phase decomposition above 0.6 wt% Zn and (ii) new findings that Zn substitution location is dependent on the concentration of Zn substitution in HA [24]. Therefore, the results of Gomes *et al.* may not be characteristic of phase pure ZnHA or ZnSiHA with lower amounts of Zn, similar to those studied here [23]. Thus, the local Zn coordination in ZnSiHA was modelled using DFT in §3.4.

### 3.2. Fourier transform infrared spectroscopy

**Figure 3.**
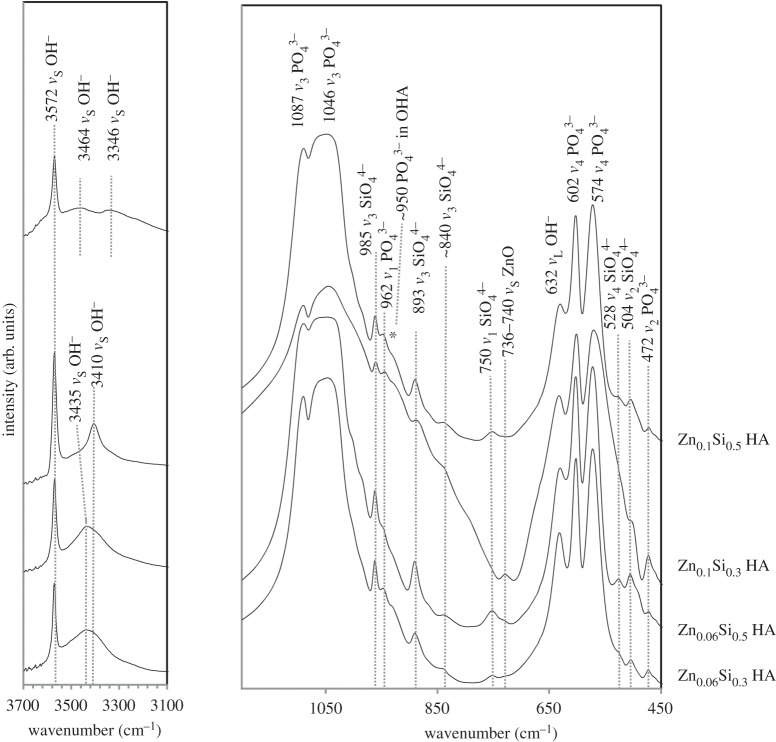
FTIR traces of calcined ZnSiHA (1100°C, 2 h, ambient atmosphere). The lower frequency phosphate/silicate region and higher frequency hydroxyl region are highlighted and major peaks are annotated.

FTIR revealed major phosphate and hydroxyl peaks typical in heat-treated HA [42] with additional features unique to Zn and Si substitution (figure 3). 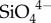 peaks highlighted by Marchat *et al.* [14] were detected for all ZnSiHA samples. An OHA peak was assigned to 950 cm^−1^ and additional hydroxyl stretching peaks (*ν*_s_) were observed in all ZnSiHA samples at high frequencies. Zn–O stretching peaks (*ν*_s_) were observed near 736–740 cm^−1^ for ZnSiHA except for Zn_0.1_Si_0.5_HA (figure 3) [43]. The two low Zn samples (Zn_0.06_Si_0.3_HA and Zn_0.06_Si_0.5_HA) had a broad peak at 3435 cm^−1^, Zn_0.1_Si_0.3_HA had a peak at 3410 cm^−1^ and Zn_0.1_Si_0.5_HA had two weak broad peaks at 3446 cm^−1^ and 3467 cm^−1^ (figure 3).

The absence of TCP or other impurity peaks agrees with the phase pure XRD spectra obtained earlier. The absence of a glassy silica peak (Si–O–Si near 798 cm^−1^) and the presence of previously reported 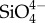 peaks support the argument that Si substituted into ZnSiHA. A weak Zn–O peak was observed near 740 cm^−1^ for Zn_0.06_Si_0.3_HA and Zn_0.06_Si_0.5_HA (740 cm^−1^), and a stronger Zn–O peak was present at 736 cm^−1^ in Zn_0.1_Si_0.3_HA (figure 3). The Zn–O peaks assigned to 736 and 740 cm^−1^ could indicate the presence of an interstitial O–Zn–O entity in the hydroxyl channel along the *c*-axis [23]. The small upshift from 736 to 740 cm^−1^ here indicates less H bonding with R–Zn–O entities, explained by lower Zn amounts in Zn_0.06_Si_0.3_HA and Zn_0.06_Si_0.5_HA samples compared with Zn_0.1_Si_0.3_HA (figure 3). Although the Zn–O IR peak was not notably shifted with Zn amount in singly substituted ZnHA (spectra not shown), the changes in H-bonding here can be attributed to mis-oriented hydroxyl ions in different configurations due to structural changes induced by silicate [14,44]. The absence of a Zn–O stretching peak for Zn_0.1_Si_0.5_HA suggests a different Zn environment in HA either where Zn–O bonds are not present (Zn on a Ca site or another interstitial site not in the hydroxyl channel), or an environment where the R*_x_*–Zn–O structure is not IR active due to symmetric stretching.

Heat-treated (and dried) ZnSiHA samples all contained additional IR hydroxyl bands that reflected changes within the hydroxyl channel. The presence of increasingly electronegative cations (Ca^2+^ versus Mg^2+^) near hydroxyl ions in nephrite has been shown to create new downshifted and split hydroxyl bands [45]. Nakamoto *et al.* showed that the high-frequency IR wavelengths depend heavily on OH–O bond distances, and shorter OH–O distances can decrease peak positions [46]. The OH–O distance in HA between two unit cells is normally too large (approx. 3.44Å) to allow for H bonding [47] and as such these shifts in the OH frequency have to be accounted for by other H-bonding entities as suggested above.

Gomes *et al.* observed a Raman peak near 3411 cm^−1^ in Zn_0.25_HA that was attributed to Zn in the hydroxyl channel at the interstitial *2b* site, and a similar peak was observed in Zn_0.06_HA, Zn_0.1_HA and Zn_0.1_Si_0.3_HA produced here (figure 3) [23,38]. The higher frequency and broader peak at 3435 cm^−1^ in Zn_0.06_Si_0.3_HA and Zn_0.06_Si_0.5_HA could be due to mis-oriented hydroxyl ions similar to those observed at 3437 cm^−1^ in HA by Park *et al.* [44], but its width obscured any peak at 3410 cm^−1^ so this does not exclude the possibility of *2b* site Zn substitution in these samples. Two broad hydroxyl peaks appeared at 3464 and 3346 cm^−1^ for Zn_0.1_Si_0.5_HA (figure 3). A broad peak was also observed in Si_0.5_HA near 3346 cm^−1^ and was attributed to altered OH–O distances from mis-oriented OH ions or OH–OPO_3_/OSiO_3_ distances. The upshifted peak at 3464 cm^−1^ is similar to the one observed by Gomes *et al.* at 3461 cm^−1^ in ZnHA, but their explanation of this peak violated their proposed ZnHA charge balance mechanism (equation (3.1)) [23]. Hu *et al.* [24] suggested that with increased Zn amounts (within the range of Zn_0.1_HA), Zn may also substitute at the CaII site. This second peak near 3464 cm^−1^ could be due to Zn substitution in a nearby CaII site with the electronegativity of Zn > Ca providing a different environment for OH–O or OH–OPO_3_/OSiO_3_ bonding. The absence of the peaks at 3410 cm^−1^ (Zn–O) suggests that Zn_0.1_Si_0.5_HA might not have Zn at the interstitial *2b* [0,0,0] site, a theory that is supported by DFT modelling in §3.4. The FTIR results suggest that multiple Zn locations may be present as substitution levels approached a critical amount in Zn_0.1_Si_0.5_HA.3.1
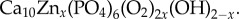


### 3.3. X-ray fluorescence

XRF performed on ZnSiHA returned values within the detection limits for CaO, P_2_O_5_, ZnO and SiO_2_ (table 3). There were slight deviations (within ±0.02) from HA stoichiometry ((Ca + Zn)/(P + Si) = 1.67) for some ZnSiHA samples (table 3), but XRD traces did not show any impurity phases and FTIR did not show peaks indicative of TCP (figures 1*a* and 3). The measured amounts of Si and Zn were lower than expected in both singly substituted SiHA and ZnHA, and co-substituted ZnSiHA. Zn measured amounts (89% ± 2 of expected Zn, *n* = 6 Zn containing samples) were closer to expected amounts compared with Si (73% ± 5 of expected Si, *n* = 6 Si containing samples) in SiHA and ZnSiHA. Differences in Zn amount were likely due to the formation of a soluble Zn–ammonia complex ion [Zn(NH_3_)_4_]^2+^*_aq_* that prevented Zn inclusion into HA during the precipitation reaction [26]. Lower Si amounts in SiHA and ZnSiHA could be explained by unintentional ambient carbonate substitution, the formation of P/Si vacancies and the incomplete hydrolysis of TEOS. Defect mechanisms in HA involving P/Si vacancies and carbonate were proposed by Palard *et al.* [15] as an explanation for deviations from ideal chemistry in SiHA. Bianco *et al.* [48] confirmed that some Si (from TEOS) and P remained behind in the mother liquor when using a wet precipitation method similar to the one used in this study. In order to retain phase purity in ZnHA (1100°C) and SiHA (1200°C) (XRD spectra not shown), the Ca amount was increased or decreased, respectively, by 1 mol% Ca compared with the calculated (Ca + Zn)/P or Ca/(P + Si) ratio based on Zn for Ca substitution or Si for P substitution. The absence of nitrogenous or carbonate species in FTIR traces suggests that a Zn–ammonia complex or significant amounts of carbonate substitution were not present in heat-treated (1100°C) ZnSiHA.

**Table 3.** XRF measurements.

	Ca/P		Ca/P + Si		Ca + Zn/P		Ca + Zn/P + Si		wt% Si		wt% Zn	
sample	expected	measured	expected	measured	expected	measured	expected	measured	expected	measured	expected	measured
HA	1.667	1.659										
Si_0.3_HA	1.750	1.752	1.667	1.693					0.85	0.54		
Si_0.5_HA	1.812	1.821	1.667	1.684					1.50	1.17		
Zn_0.06_HA	1.656	1.663			1.667	1.674					0.40	0.37
Zn_0.1_HA	1.650	1.657			1.667	1.673					0.66	0.58
Zn_0.06_Si_0.3_HA	1.743	1.724	1.656	1.655	1.754	1.734	1.667	1.665	0.84	0.61	0.41	0.35
Zn_0.06_Si_0.5_HA	1.820	1.794	1.657	1.651	1.832	1.805	1.667	1.661	1.52	1.22	0.40	0.36
Zn_0.1_Si_0.3_HA	1.737	1.739	1.650	1.669	1.755	1.756	1.667	1.685	0.85	0.60	0.65	0.56
Zn_0.1_Si_0.5_HA	1.811	1.803	1.650	1.674	1.829	1.821	1.667	1.690	1.50	1.09	0.66	0.59

Interestingly, the quantities of Si and Zn incorporated into ZnSiHA were similar to those in SiHA and ZnHA (table 3). The equal and opposite calcium changes required in singly substituted ZnHA and SiHA explain why ZnSiHA did not require Ca/P ratio alterations through the addition or subtraction of 1 mol% Ca compared with calculated Ca/P ratios (table 1). A phase pure ZnSiHA product was obtained despite the variances in Si and Zn from expected values (table 3 and figures 1*a* and 3). This suggests that equation (2.1) may not accurately describe the charge balance mechanism for Zn and Si insertion into ZnSiHA despite the production of a phase pure product. In particular, at our higher zinc concentrations (Zn_0.1_Si_0.3_HA and Zn_0.1_Si_0.5_HA), the (Ca + Zn)/(P + Si) ratio increases to 1.685 and 1.690, respectively. This may indicate substitution into the *2b* or alternative interstitial position (see §3.4), which would be in agreement with Hu *et al.* [24] for their lower Zn concentration (0.1 mol%) in ZnHA. However, these small deviations from the (Ca + Zn)/(P + Si) ratios were within 1% of expected values, which was also lower than the mol% of substituted Zn.

### 3.4. Computational modelling

**Figure 4.**
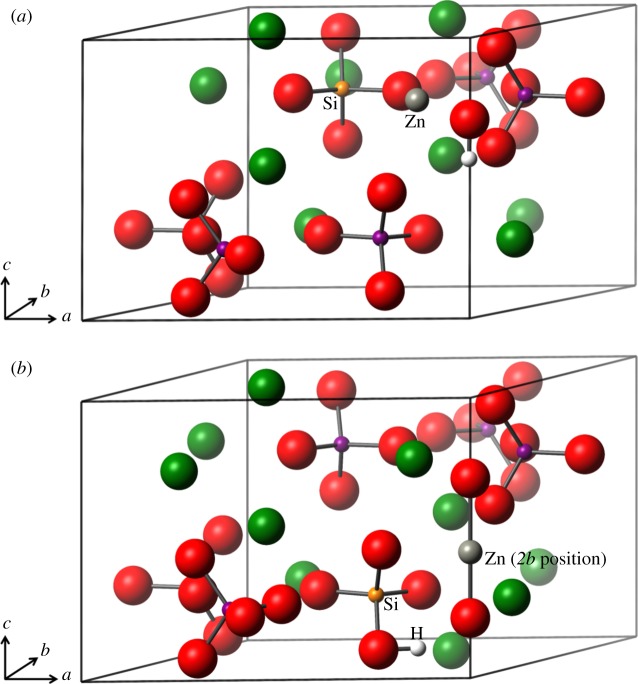
(*a*) Silicate substitution and a zinc ion replacing a calcium ion. One hydroxyl ion has been removed from the *c*-axis for charge compensation. (*b*) Silicate substitution and a charge compensatory H atom positioned on the silicate ion. The zinc substitution is on the *c*-axis in the *2b* position between two oxygen ions. Oxygen is shown in red, calcium green, phosphorus purple, zinc grey, silicon orange and hydrogen in white. (Online version in colour.)

To allow for charge compensation, two basic models were created. In both cases, a phosphate ion is substituted out and replaced with a silicate ion. There were only minor differences in formation energy between the six phosphorus substitution sites for the silicate ion, but the lowest energy position was nevertheless chosen for the double substitution. In the first basic model, in addition to the silicate substitution (with concomitant hydroxyl ion removal), a calcium ion is substituted out and replaced by a zinc ion. All 10 calcium substitution positions were interrogated. Figure 4*a* shows the zinc in one of the type II positions.

In the second general case, a full complement of calcium ions was retained and a zinc ion at the *2b* position (on the *c*-axis) referred to earlier was created. In this conformation, both hydrogen atoms were removed from the hydroxyl ions and the initial position for the zinc ion was in between the two oxygen ions on the *c*-axis as shown in figure 4*b*. However, to retain overall charge balance of the cell, one hydrogen atom was required in the cell. Two possibilities were examined, one with a protonated phosphate ion and one with a protonated silicate ion. This second conformation is shown in figure 4*b*.

The formation energies for all the conformations are given in table 4. As can be seen from table 4, the lowest energy configurations are those with the zinc ion in the *2b* starting configuration. Of the other configurations, substitution of Ca1 gave the lowest formation energy. It is of note that although the lowest formation energies are of type II calcium ions, there is no clear distinction between formation energy and site type, as is usually the case with single substitutions [22,49]. This shows that the interaction between the silicate and zinc ions has an energetic effect large enough to distinguish the calcium sites as unique potential substitution positions. In general, the formation energies are positive but small, suggesting that the substitution at this concentration is unfavourable but only marginally so. This is not an unexpected result given that experimentally the substitution can be made but at rather lower concentrations than calculated here.

**Table 4.** The formation energies, *E*_f_, of the double SiZn substitution in order of favourability.

Zn location	*E*_f_ (eV)	site type
interstitial SiO_4_H	1.2017	—
interstitial PO_4_H	1.3761	—
Ca 1	2.3427	II
Ca 2	2.4441	II
Ca 3	2.5324	II
Ca 4	2.6081	II
Ca 5	2.6896	I
Ca 6	2.691	I
Ca 7	2.802	II
Ca 8	2.8572	I
Ca 9	2.9149	II
Ca 10	2.9216	I

**Figure 5.**
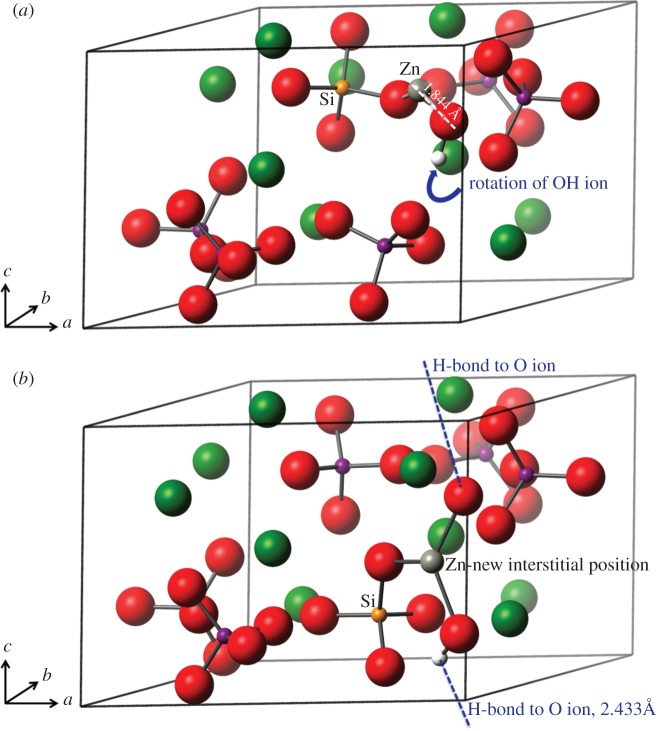
(*a*) Silicate substitution and a zinc ion replacing a calcium ion. One hydroxyl ion has been removed from the *c*-axis for charge compensation. The zinc ion is bonded to the hydroxyl ion oxygen atom and one of the silicate ion oxygen atoms. The hydroxyl ion has been pulled off the *c*-axis. (*b*) Silicate substitution with a charge compensatory H atom positioned on the silicate ion. The zinc substitution is on the *c*-axis between two oxygen ions. The hydrogen atom has reattached to one of the *c*-axis oxygen atoms and the zinc is strongly bonded to both the *c*-axis oxygen atoms. Oxygen is shown in red, calcium green, phosphorus purple, zinc grey, silicon orange and hydrogen in white. (Online version in colour.)

Figure 5*a* shows the final relaxed structure of the Ca1 substitution. It is clear that there is a rotation of the hydroxyl ion, away from the *c*-axis and towards the zinc ion.

The final structures of the *2b* unit cells are considerably different from the starting configurations, with the hydrogen atom having rejoined one of the *c*-axis oxygen ions and the zinc having moved away from the *c*-axis to an interstitial position. This is shown in figure 5*b*. This mis-orientation of the hydroxyl ions in these models, particularly in the interstitial position, may be reflective of the small upshift in the 736 cm^−1^ band and the additional hydroxyl IR bands described in §3.2 (figure 3), which was attributed to a reduction in hydrogen bonding with the R–Zn–O entity. Indeed, the OH–O distance has increased in the interstitial model to 2.950 Å from the phase pure HA distance of 2.433 Å, hence a weakening of the hydrogen bonding. These results may explain the OH peaks of varying IR frequencies discussed above (§3.2).

The lattice parameters of the most favourable sites for both the *2b* position cells (SiO_4_H) and the regular calcium substitution positions (Ca1) are presented in table 5 along with the experimental results for comparison. It should perhaps be noted that the increase in cell volume between the experimental parameters and the theoretical Ca1 parameters is accounted for in changes to the unit cell angles. In both *2b* and the Ca1 models, there is an increase in the *c* parameter of between 1.13% and 1.6% as compared with optimized phase pure HA, which is very similar to the experimentally derived values (§3.1). In the Ca1 model, the *a* parameter decreases marginally (−0.2%), while in the *2b* model there is an *a* parameter increase, in line with our experimental results. However, contrary to the experimental determination of the lattice parameters, in the theoretical calculations the unit cell was not constrained, hence *a* ≠ *b*. Indeed, the *b* parameters in both models increased from the phase pure HA value (by 1.75% for the *2b* model and 0.05% for the Ca1 model). The lattice parameters of the Ca1 substitution most closely match those of the experimental work (within 1%), but even the SiO_4_H interstitial values match within 2.4%, showing good agreement.

**Table 5.** Lattice parameters for the SiO_4_H and Ca1 substitution cells in comparison with the experimental values.

configuration	*a* (Å)	*b* (Å)	*c* (Å)	volume (Å^3^)
theoretical—SiO_4_H	9.53	9.6437	6.9281	543.94
theoretical—Ca1	9.4575	9.482	6.9611	529.7
experimental	9.4265	9.4265	6.9009	531.05
% difference SiO_4_H—Exp.	−1.1	−2.3	−0.6	−2.4
% difference Ca1—Exp.	−0.3	−0.6	−0.9	1

The distribution of electron density, calculated by Mulliken population analysis [50], shows that bonding between the zinc ions and oxygen has a much stronger covalent character than found between the calcium ions and oxygen atoms. In the Ca1 cell, the bond populations of the Zn–O bonds are an average of 0.36 |*e*| and in the SiO_4_H structure 0.46 |*e|*. Typically, the Ca–O bond populations are of the order of 0.05–0.18 |*e|*. Of particular note, in the SiO_4_H structure, the Zn–O bond length to the sole *c*-axis oxygen ion is 1.844 Å with a bond population of 0.60 |*e|*. This bond population is as strong as those between phosphorus and oxygen within single PO_4_ ions and can be regarded as a covalent bond. While it might be expected that the OH–O bond distance will have decreased with the Zn on the *c*-axis, this is actually not what occurs due to the movement of the Zn ion off the *c*-axis to the truly interstitial position (figure 5*b*) and the twisting of the reformed OH ion. In the optimized HA unit cell, the OH–O distance is calculated to be 2.433 Å, and in the SiO_4_H cell 2.778 Å.

For comparison with previous results of the author and others [21,22,51,52], the formation energy of the zinc substitution on the *2b* site was calculated for the singly substituted ZnHA. The formation energy for this substitution was 1.72 eV. This compares with zinc substitution in the CaI site (4.82 eV) and CaII site (4.60 eV). In the previous work by the author [22], the *2b* site had not been considered as a possible location for the zinc substitution, which makes this result an interesting addition to this work. It is also worth noting that the *c* parameter increases from phase pure HA by 1.5% even though the oxygen atoms are brought closer together by the Zn ion between them. We hypothesize that this expansion in the *c*-axis is largely due to the breaking of the hydrogen bonding between adjacent hydroxyl ions of the same unit cell. For illustration, the resulting zinc position is very similar to the starting zinc position of figure 4*b*, without any movement off the *c*-axis, as occurs in the ZnSiHA model.

## 4. Conclusion

A wet precipitation method for producing ZnSiHA with Zn amounts up to 0.6 wt% and Si amounts up to 1.2 wt% was described here for the first time. Upon heating to 1100°C, these products remained phase pure as measured with XRD. Co-substitution of Zn and silicate in HA was evidenced by lattice parameter expansions unique to ZnSiHA compared with singly substituted materials; silicate- and Zn-related FTIR peaks; and quantitative elemental analysis of Ca, P, Zn and Si with XRF. Zn induced changes to the hydroxyl stretching region in ZnSiHA samples and variations in expected Zn and Si amounts suggested that the assumed isoelectronic substitution mechanism of Zn for Ca does not accurately describe ZnSiHA wet precipitation synthesis. DFT modelling tested Zn substitution at Ca sites and a *c*-axis position associated with Zn substitution in ZnHA. Interestingly, the lowest energy Zn location was at a new interstitial position (figure 5*b*) just off the *c*-axis near a silicate anion in ZnSiHA, which helped to explain our FTIR results in particular. Experimental and computed lattice parameters were within 2.4%. X-ray near-edge structures analysis studies would be useful to experimentally follow Zn coordination in ZnSiHA at different Zn concentrations in future studies, especially as we can now expect an interstitial substitution at some concentrations, which was not previously suspected. These findings provide a foundation for future production and characterization of ZnSiHA. Such a material could be used to deliver an increased amount of Zn as an anti-microbial agent while retaining the desirable effects of silicon on bone formation.

The complex nature of bone/biomaterial interaction makes it difficult to assign a single mechanism to the success of a given ionic substitution in HA. Atomic changes induced by ionic substitution into HA can translate into complex changes in microstructure, lattice solubility and surface charge. Any of these changes can impact the bioactive bone response. The mechanism of action for these materials could be due to either an active (ionic release) and/or passive (bound atoms, altered surface charge, protein adsorption, etc.) phenomenon [10]. For example, higher concentrations of triple-point boundaries in SiHA compared with HA have been suspected as the driver of enhanced lattice solubility [11,53], and SiHA has been shown to have an altered surface charge compared with HA [54,55]. Future work that investigates the micro- and macro-structural changes resulting from Zn and silicate co-substitution in HA will help in the interpretation of the biological response to ZnSiHA that has yet to be reported. These Zn, Si co-substituted materials, when used as a synthetic aid to bone regeneration, could potentially provide unique biological solutions to problems currently unsolved by more traditional, singly substituted HA.

## Data accessibility

Supplemental data including XRD traces of phase impure ZnSiHA (table 1), XRD traces used in Rietveld refinement of lattice parameters, FTIR traces, XRF oxide raw data and chemical structures (CIF and CASTEP files) are available in the Dryad Digital Respository (http://dx.doi.org/10.5061/dryad.306g8).
